# Imiquimod Targets Toxoplasmosis Through Modulating Host Toll-Like Receptor-MyD88 Signaling

**DOI:** 10.3389/fimmu.2021.629917

**Published:** 2021-03-09

**Authors:** Maguy Hamie, Rania Najm, Carine Deleuze-Masquefa, Pierre Antoine Bonnet, Jean-François Dubremetz, Marwan El Sabban, Hiba El Hajj

**Affiliations:** ^1^Department of Experimental Pathology, Microbiology and Immunology, Faculty of Medicine, American University of Beirut, Beirut, Lebanon; ^2^IBMM, Université de Montpellier, CNRS, ENSCM, Montpellier, France; ^3^UMR 5235 CNRS, Université de Montpellier, Montpellier, France; ^4^Department of Anatomy, Cell Biology and Physiological Sciences, Faculty of Medicine, American University of Beirut, Beirut, Lebanon

**Keywords:** cerebral toxoplasmosis, acute toxoplasmosis, cytokines, reactivation, pathogen-host interaction

## Abstract

*Toxoplasma gondii* is a prevalent parasite of medical and veterinary importance. Tachyzoïtes and bradyzoïtes are responsible for acute and chronic toxoplasmosis (AT and CT), respectively. In immunocompetent hosts, AT evolves into a persistent CT, which can reactivate in immunocompromised patients with dire consequences. Imiquimod is an efficient immunomodulatory drug against certain viral and parasitic infections. *In vivo*, treatment with Imiquimod, throughout AT, reduces the number of brain cysts while rendering the remaining cysts un-infectious. Post-establishment of CT, Imiquimod significantly reduces the number of brain cysts, leading to a delay or abortion of reactivation. At the molecular level, Imiquimod upregulates the expression of Toll-like receptors 7, 11, and 12, following interconversion from bradyzoïtes to tachyzoïtes. Consequently, MyD88 pathway is activated, resulting in the induction of the immune response to control reactivated *Toxoplasma* foci. This study positions Imiquimod as a potent drug against toxoplasmosis and elucidates its mechanism of action particularly against chronic toxoplasmosis, which is the most prevalent form of the disease.

## Introduction

*Toxoplasma gondii* (*T. gondii*) is the etiologic agent of toxoplasmosis, a common human and zoonotic disease. *T. gondii* infects ~30% of the world's human population (1), with a sero-prevalence that can reach 80% in some regions (2). Recently, toxoplasmosis was classified by the Centers for Disease Control and Prevention as a neglected parasitic infection, requiring public health action (3). Toxoplasmosis manifests as acute and chronic forms (AT and CT, respectively). In immunocompetent hosts, AT is caused by the presence of replicating tachyzoïtes, which convert in the brain and the skeletal muscles into persistent bradyzoïte cysts [(4, 5), reviewed in Wohlfert et al. (6), Poncet et al. (7)].

While toxoplasmosis is considered asymptomatic in most immunocompetent patients, several medical conditions are associated with *T. gondii* infection (8). Anxiety, depression and suicide attempts correlate with seropositivity to parasite antigens (9, 10). Higher antibody titers against *T. gondii* are also reported in different types of cancers (11–13), particularly in brain cancers due to the ability of the parasite to interfere with the brain cells miRNAome (14). Furthermore, CT promotes the progression of several neuropathies such as schizophrenia and Parkinson disease (15–17). In immunocompromised patients, reactivation of CT associates with severe morbidity and mortality (18–20). Reactivation usually occurs in HIV patients or in those who receive immunosuppressive therapies in the context of bone marrow and solid organ transplantation, or chemotherapy against cancer (21–24).

Treatment of toxoplasmosis remains limited to general anti-parasitic/anti-bacterial drugs. These include spiramycin, azithromycin, atovaquone, pyrimethamine-sulfadiazine, pyrimethamine-clindamycin, and trimethoprim-sulfamethoxazole [reviewed in Dard et al. (25)]. The recommended first-line therapy remains the combination of pyrimethamine, an inhibitor of the dihydrofolate reductase enzyme, with sulfadiazine, an inhibitor of the dihydropteroate synthase [reviewed in Montoya and Liesenfeld (20), Lapinskas and Ben-Harari (26)]. However, this combination suffers from several limitations, including hematological side effects (27), elevation in serum creatinine and serum liver enzymes, allergic reactions (28) and emergence of resistant parasites (29). In addition, these drugs, whether given as prophylactic or therapeutic agents, target only AT and remain ineffective against CT (30–33).

Imiquimod is an immune response modulating drug, used for the treatment of some viral infections (34, 35). This drug proved potent against a parasitic infection causing cutaneous leishmaniasis (36–38). Recently, Imiquimod demonstrated anti-*Toxoplasma* effects *in vitro*, and *in vivo* using a type I virulent strain. In addition, mice prophylactically pre-treated with imiquimod, before infection, showed prolonged survival time compared with post-treated and control groups, highlighting a prophylactic potency of this drug against toxoplasmosis (39). Imiquimod binds Toll-like receptor-7 (TLR-7) (40, 41), and activates the innate immune response through MyD88 signaling pathway (41). In mammals, TLR family of proteins comprises thirteen members (TLR1-13) (42), which are transmembrane receptors localized on the plasma membrane and endosomal/lysosomal cellular compartments. TLRs recognize distinct pathogenic constituents (43), to couple innate to adaptive immunity (44). Following infection with *T. gondii*, TLRs expressed on innate immune cells detect the parasite (45). Murine TLR-11 and 12 of DCs, recognize Profilin, a *Toxoplasma* Pathogen-associated molecular pattern (PAMP) [(46–48), reviewed in Poncet et al. (7)], to primarily signal through the adaptor protein MyD88 (49), leading to the activation of several signaling pathways including nuclear factor kappa-light-chain-enhancer of activated B cells (NF-κB), and mitogen-activated protein kinase (MAPK). This subsequently mounts the protective immune response through the production of interleukin-12 (IL-12), Interferon-gamma (IFN-γ), among others (47, 50). TLR-11/TLR-12 dimer is important for DC response and IL-12 production (46, 51). Indeed, TLR-11^−/−^ mice display low levels of IL-12 production (47, 52), and TLR-12^−/−^ results in a loss of resistance to toxoplasmic infection, as well as a deficiency of the gene encoding MyD88 (46, 52). Profilin was proved indispensable for invasion and active egress of *T. gondii* from cells (53–55). Parasites lacking *T. gondii* Profilin but expressing the Profilin of another apicomplexan parasite, *Plasmodium falciparum*, exhibit a restored invasion and egress, but remain unable to induce the activation of TLR11/12, thus abrogating their TLR-11-dependent production of IL-12 both *in vitro* and *in vivo* (55).

We assessed the potency of Imiquimod against AT and CT. Imiquimod treatment reduced tachyzoïte replication *in vitro*. Treatment of mice, during AT, resulted in the recruitment of T cells to the peritoneum and spleen, and significantly decreased the number of brain cysts upon establishment of CT. Remarkably, the remaining brain cysts from Imiquimod treated mice, failed to establish a new infection. We also investigated the efficacy of Imiquimod against CT, the most common form of toxoplasmosis. Imiquimod sharply reduced the number of brain cysts, and delayed or abrogated their reactivation in an established CT. TLR-7, 11, and 12 expression was significantly increased in the brains of treated mice following an Imiquimod-induced interconversion. TLRs upregulation activated MyD88-MAPK pathway and induced the subsequent activation of the immune response. *In vitro*, a *Toxoplasma* knockout strain for Profilin, does not respond to Imiquimod treatment, suggesting a potential effect of this drug on Profilin/TLR-11/12 interaction. This study repositions Imiquimod against another parasitic disease, and proves it as an effective treatment for toxoplasmosis. Furthermore, it elucidates the mechanism of action of this drug, especially against the chronic phase, which the most prevalent from of the disease. Resolving toxoplasmosis will positively impact the parasite-associated diseases, both at the health and economic levels.

## Materials and Methods

### Preparation of Drugs

Imiquimod was purchased from Molekula (Wessex House, Shaftesbury, Dorset, UK). The original stock was prepared in dimethylsulfoxide (DMSO) at the concentration of 0.1 M and stored at −80°C. For all the *in vitro* experiment, working solutions were freshly prepared in culture media at the concentration of 1 μM. For the *in vivo* experiments, Imiquimod was dissolved in DMSO and diluted in an equal volume of lipofundin-MCT/LCT 20% emulsion (B. Braun Melsungen, AG/ D-34209 Germany) before its intraperitoneal administration to the mice.

### Parasite Lines and Mammalian Cell Cultures

*Toxoplasma gondii* 76K strain was provided by Dr. Mathieux Gissot. Profilin knockout strain ΔTg*PRFe*/Tg*PRFi* and its control RH*TATi-1* were provided by Dr. Dominique-Favre Soldati. Tachyzoïtes were serially passaged in human foreskin fibroblasts (HFFs) (American Type Culture Collection (ATCC)-CRL 1634) cultured in Dulbecco's Modified Eagle's Medium (DMEM) (GIBCO, Invitrogen) and supplemented with 10% of fetal bovine serum (FBS), 1% penicillin-streptomycin, and 1% glutamine.

Peritoneal macrophages were harvested from BALB/c mice, following their induced recruitment by thioglycollate (38.5 g/L, Sigma). After peritoneal lavage, cells were collected by centrifugation. One million murine macrophages were seeded in 6-well-plates and cultured in RPMI medium supplemented with 10% FBS, 1% penicillin-streptomycin, and 1% glutamine (Invitrogen).

To compare Imiquimod to clinically used drugs, cells were infected with the 76K strain at 1:3 parasites to macrophage ratio for 24 h, prior to their treatment with 1 μm of Imiquimod, 0.4 μm of Pyrimethamine, 1.6 μm of Sulfadiazine (56), or the combination of Pyrimethamine and Sulfadizaine for 48 h.

To examine whether Imiquimod signals through TLR11/12, cells were infected with RH*TATi-1* and ΔTg*PRFe*/Tg*PRFi* at 1:3 parasite to macrophage ratio for 24 h, prior to their treatment with 1 μm of Imiquimod for 48 h.

### *In vitro* Interconversion From Tachyzoïtes to Bradyzoïtes

Confluent HFF cells cultured in a 6-well plate (500,000 cells per well) were infected with 1,000 tachyzoïtes of the 76K strain/well. After 24 h of incubation in complete DMEM medium under 5% CO_2_, cells were maintained in induction medium (RPMI 1640 without NaHCO_3_, HEPES 50mM, 3% FBS, pH 8.2) and in absence of CO_2_. The basic medium was changed every other day to maintain the pH at 8.2. After 10 days, cells infected with bradyzoïtes were treated every other day with 1 μm of Imiquimod. On day 14, infected cells were harvested for western blot and immuno-fluorescence assays.

### Bradyzoïte Quantification by Immunofluorescence Assay

Bradyzoïte conversion was confirmed by staining the cyst wall with Biotinylated *Dolichos biflorus* lectin (DBA) (57). Following *in vitro* switch, infected cells with cysts of 76K, were fixed with 4% paraformaldehyde in PBS for 20 min, permeabilized in 0.2% Triton for 10 min, blocked with 10% FBS in PBS for 30 min. T_8_2C_2_ primary monoclonal antibodies directed against P34 (stock concentration 2 mg/ml, dilution 1:500) (58) were used to stain bradyzoïtes. Biotinylated DBA (Sigma, Cat. NoB-1035) was used at the dilution of 1:100. Anti-mouse secondary antibody (Abcam, ab150116) was used at the concentration of 1:500 (stock concentration 2 mg/ml, dilution 1:500). Streptavidin (Sigma) was used at the dilution of 1:100. Coverslips were mounted on slides using a Prolong anti-fade kit (Invitrogen, P36930). Z-section images were acquired by confocal microscopy using confocal microscopy (Zeiss LSM 710) and all images were analyzed using Zeiss Zen software.

### Protein Expression Analysis

#### Western Blot

Proteins from various experimental procedures were separated on polyacrylamide gels with different percentages according to the molecular weight of desired proteins and transferred to nitrocellulose membranes (BIO RAD Cat# 162-0112). Membranes were probed with different primary antibodies followed by anti-mouse (m-IgGk BP-HRP, sc-516102) or anti-Rabbit (Mouse anti-rabbit IgG-HRP, sc-2357) (Santa Cruz, 1:5000) secondary antibodies conjugated to Horseradish peroxidase (HRP). Bands were visualized using luminol chemiluminescent substrate (Bio-Rad, Cat# 170-5061).

Primary antibodies used in our study are: T_8_ 3B_1_ primary monoclonal antibody directed against p18 (2mg/ml) (1:1000) (58, 59), T_4_ 1E_5_ monoclonal antibody directed against P30 (2mg/ml) (SAG-1/1:1000) (58), TLR-7, 11, 12 polyclonal antibodies Thermofisher (Cat#PA5-11605; 1:500; Cat# PA1-41080; 1:1000; Cat# PA1-41037; 1:500, respectively), MyD88 monoclonal antibody Abcam (Cat#ab135693; 1:1000), total ERK1/2 (*137F5)* Rabbit polyclonal cell signaling (Cat#*4695*; 1:1000), Phospho–P44/42 MAPK ERK1/2 (Thr202/Tyr204) Rabbit polyclonal antibody cell signaling (Cat#4397; 1:1000), β-Actin Mouse cell signaling (Cat#8H10D10; 1:1000), GAPDH antibody conjugated to HRP from Abnova (Cat#MAB5476; 1:20000).

#### Enzyme-Linked Immunosorbent Assay (ELISA)

Brains from infected BALB/c mice with 76K were harvested after 2, 3, or 4 weeks of treatment with 50 μg of Imiquimod/mouse. Following brain homogenization, supernatants were collected, and ELISA was performed using Single Analyte ELISArray Kit (Qiagen). Briefly, supernatants were spun for 10 min at 1,000 g, transferred to new Eppendorf tubes, and diluted using a specific cocktail of antigens (IL-12, IL-1β, and IFN-γ) provided by the kit. Samples were then transferred to ELISA plate, and were incubated for 2 h. After three washes, the detection antibody was added and incubated for 2 h, followed by Avidin-HRP addition for 30 min. Wells were washed and developed in the dark for 15 min, before addition of the stop solution. Optical density was recorded at 450 and 570 nm.

#### Immune Cell Identity Staining by Flow Cytometry

BALB/c mice were infected with 1,000 tachyzoïtes (60) and treated with 50 μg of Imiquimod on days 2 and 3 and sacrificed on day 4 post-tachyzoïtes injection. Peritoneal lavage was performed. Cells recovered from the peritoneum and spleen-derived murine cells from untreated or treated BALB/c mice, were stained for 20 min in dark, with anti-CD11b (BD Biosciences, 2 μg/mL, lot number: 553311) or anti-CD3 (BD Biosciences, 2 μg/mL, lot number: 561799), directly conjugated to Phycoerythrin (PE). Labeled samples were washed twice and 5,000 events were acquired using a Guava flow cytometer easy Cyte TM 8 (Millipore).

#### Transcriptional Expression Analysis

Quantitative Real Time PCR (RT-qPCR) was performed using CFX96 (Biorad). Primers to detect different transcripts in the brains of BALB/c infected mice, are listed in (Table 1). Glyceraldehyde-3-Phosphate dehydrogenase (GAPDH) was used as housekeeping gene (Table 1). In qRT-PCR, individual reactions were prepared with 0.25 μM of each primer, 150 ng of cDNA and SYBR Green PCR Master Mix to a final volume of 10 μl. PCR reaction consisted of a DNA denaturation step at 95°C for 3 min, followed by 40 cycles (denaturation at 95°C for 15 s, annealing at the appropriate temperature of the used primers for 60 s, extension at 72°C for 30 s). For each experiment, reactions were performed in duplicates and the expression of individual genes was normalized to GAPDH threshold cycle (Ct) values. The Threshold cycle (Ct) corresponds to the cycle at which there is a significant detectable increase in fluorescence. Data were plotted by calculating ΔCt (Ct_target gene_ − *Ct*_GAPDH_). Thereafter, ΔΔCt is calculated according to the Livak method: 2^−ΔΔCt^ to obtain the percentage of expression (61).

**Table 1:** Summary of primers used for Real-time quantitative PCR.

**Primer**	**Sequence 5′ → 3′**	**Annealing T^0^C**
Mouse GAPDH Forward Primer	5′-CATGGCCTTCCGTGTTCCTA-3′	59.4
Mouse GAPDH Reverse Primer	5′-CCTGCTTCACCACCTTCTTGAT-3′	60.3
SAG-1 Forward primer	5′-ACT CAC CCA ACA GGC AAA TC 3′	56.5
SAG-1 Reverse primer	5′- GAG ACT AGC AGA ATC CCC CG-3′	56.6
BAG-1 Forward primer	5′-GCGGAGAAAGTGGACGATGATGG-3′	62
BAG-1 Reverse primer	5′-GTCGGGCTTGTAATTACTCGGG-3′	62
TLR-11 Forward primer	TCCTTCCTCTGATTAGCTGTCCTAA	57
TLR-11 Reverse primer	TCCACATAATTTCCACCAACAAGT	57
TLR-12 Forward primer	GCCGCCATTCCAAGCTATC	57
TLR-12 Reverse primer	CTCCACAGTCCGAGGTACAACTT	57
TLR-7 Forward primer	TTCCTTCCGTAGGCTGAACC	57
TLR-7 Reverse primer	GTAAGCTGGATGGCAGATCC	57
Mouse CXCL9 Forward Primer	5′-TGT GGA GTT CGA GGA ACC CT-3′	60.5
Mouse CXCL9 Reverse Primer	5′-TGC CTT GGC TGG TGC TG-3′	57.2
Mouse CXCL10 Forward Primer	5′-AGA ACG GTG CGC TGC AC-3′	57.2
Mouse CXCL10 Reverse Primer	5′-CCT ATG GCC CTG GGT CTC A-3′	61.7

### *In vivo* Studies

#### Acute Toxoplasmosis

To determine the effective dose of Imiquimod, 8 to 10 weeks old female BALB/c mice (5 mice per condition) were intraperitoneally injected with 250 parasites of 76K, an optimized dose capable of establishing CT following AT, without lethality. Mice were then treated intraperitoneally with 2.5 mg/kg/day (50 μg per mouse) or 5 mg/kg/day (100 μg per mouse) of Imiquimod every other day, from day 2 until day 8 and mice were sacrificed on day 10 to assess tachyzoïte burden in the spleen.

To test the effect of Imiquimod on immune cell recruitment during AT, 8 to 10 weeks old female BALB/c mice (5 mice per condition) were intraperitoneally injected with 1,000 parasites of 76K as described (60). Control mice were treated with sulfadiazine (200 mg/L administered in drinking water). Imiquimod treatment was performed on days 2 and 3 and mice were sacrificed on day 4 p.i.

To test the potency of Imiquimod on brain cyst formation, 8 to 10 weeks old female BALB/c mice (10 mice per condition) were intraperitoneally injected with 250 parasites of 76K, and treated during AT, with 2.5 mg/kg/day of Imiquimod from day 4 until day 32. Mice were then sacrificed, brains were harvested, and cysts were extracted using an optimized Percoll method (GE Healthcare Percoll Bio-Sciences AB Lot 10221921) (62, 63). To test the viability of the remaining cysts found in the brains of treated mice, 20 cysts were orally inoculated by gavage into new BALB/c mice (10 mice per condition) and left for survival.

#### Chronic Toxoplasmosis

To explore the effect of Imiquimod on CT that start at day 21 post infection (p.i.), 8 to 10 weeks old female BALB/c mice (18 mice per condition) were orally infected by gavage, with 20 cysts of 76K. Chronically infected mice were treated with 2.5 mg/kg/day of Imiquimod, every other day, and starting day 21 and until day 49 p.i. (Timeline described in 2C). On day 49 p.i., ten mice were sacrificed and brain cysts were counted following an optimized percoll extraction method (62, 63). To test the viability of the remaining cysts found in the brains of treated mice and their capacity of reactivation, the remaining mice (eight mice per condition) were treated with Dexamethasone (5 mg/L) (64) and assessed for survival for more than 120 days.

To assess the upregulation and the time point at which TLR-7, 11, 12 and are upregulated, 8 to 10 weeks old female BALB/c mice (5 mice per condition) were intraperitoneally injected with 250 parasites of 76K. Chronically infected mice were treated with 2.5 mg/kg/day of Imiquimod, every other day, starting day 21 and until day 49 p.i. Mice were sacrificed on a weekly basis (days 35, 42, and 49 p.i.). Total brains were harvested and TLR-7, 11, 12 and transcript levels were assessed by RT-qPCR. All remaining molecular studies on *in vivo* treated mice, were performed on brains of mice sacrificed at day 49 p.i.

### Ethics Statement

All mice protocols were approved by the Institutional Animal Care and Utilization Committee (IACUC) of the American University of Beirut (AUB) (Permit Number: #18-02-461). All animals were housed in specific pathogen free facility with a 12 h ON/OFF light cycle. Humane endpoints were fully respected as per AUB IACUC following Association for Assessment and Accreditation of Laboratory Animal Care International guidelines and guide of animal care use book (Guide, NRC 2011). Mice were monitored on a daily basis. To verify the acute phase of the infection, blood was withdrawn following deep anesthesia with isoflurane by inhalation. Mice were sacrificed if any abnormal ethical features are noticed.

### Statistics

The experiments were analyzed using either two-tailed Student's *t*-tests or Anova one way to determine the statistical significance of differences observed between indicated groups for parametric comparisons and presented as averages with standard deviations. Statistical significance is reported as ^*^ for *P*-value between 0.05 and 0.01, ^**^ for *P*-value between 0.01 and 0.001, and ^***^ for *P*-value < 0.001.

## Results

### Imiquimod Is Effective on *Toxoplasma gondii* Tachyzoites *in vitro* and *in vivo*

**Figure 1:**
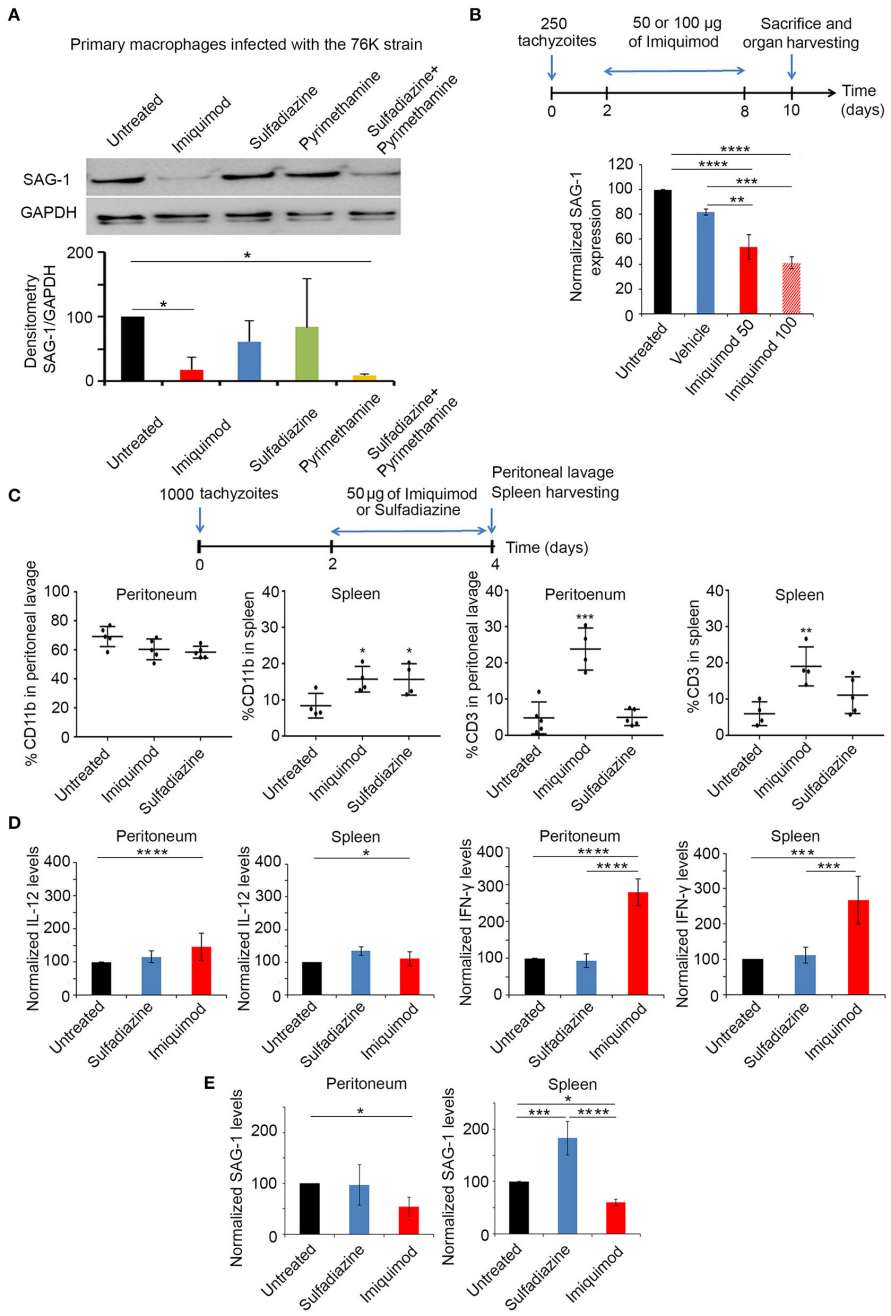
Imiquimod exhibits a potent effect against acute toxoplasmosis. **(A)** Western Blot analysis for SAG-1 expression in primary macrophages infected with the 76K without or after treatment with Imiquimod, Sulfadiazine, Pyrimethamine, or the combination of Sulfadiazine/Pyrimethamine. The results depict one representative experiment among three independent ones. **(B)** Timeline schedule for assessment of Imiquimod efficacy on tachyzoïtes *in vivo*. On day 0, BALB/c mice were injected with 250 tachyzoïtes/mouse of 76K, on day 2 post injection mice were treated every other day with Imiquimod (50 or 100 μg/mouse) or the vehicle (Lipofendin + DMSO). At day 10, spleens were harvested and quantitative Real-Time PCR for SAG-1 (five mice per condition) was performed. SAG-1 expression was normalized to GAPDH. The results are expressed as percentage of untreated control (±) SD. The Anova one-way test was performed to validate significance. *, **, and *** indicate *p*-values ≤ 0.05; 0.01; and 0.001, respectively. *P*-values less than 0.05 were considered significant. **(C)** Timeline schedule for immune cell recruitment. Briefly, on day 0, BALB/c mice were injected with 1,000 tachyzoïtes/mouse of 76K (five mice per condition); on day 2 and 3 mice were treated either with Imiquimod (50 ug/mouse) or with sulfadiazine (200 mg/L in drinking water). At day 4, spleens and peritoneal lavage were harvested (five mice per condition) and analyzed by Flow cytometry **(C)** showing the percentage of CD11b and CD3 as indicated and by quantitative Real-Time PCR for IL-12, IFN-γ and SAG-1 as indicated **(D,E)**. The Anova one-way test was performed to validate significance. *, **, and *** indicate *p*-values ≤ 0.05; 0.01 and 0.001, respectively. *P*-values less than 0.05 were considered significant.

We investigated the potency of Imiquimod on macrophages infected with tachyzoïte stage of the 76K strain of *T. gondii*. Primary-elicited Macrophages were infected at 1:3 parasite to cell ratio. Twenty-four hours p.i., cells were treated with 1 μm of Imiquimod, 0.4 μm of Pyrimethamine, 1.6 μm of Sulfadiazine, or the combination of Pyrimethamine and Sulfadiazine for 48 h. Imiquimod significantly reduced the expression of the tachyzoïte-specific protein surface antigen 1 (SAG-1) (Figure 1A). This decrease was more prominent than Sulfadiazine or Pyrimethamine single agents, and was even better than the combination of these two clinically used drugs against AT (Figure 1A). This finding proves the anti-parasitic efficacy of this drug on tachyzoïtes *in vitro*, and highlights its increased potency as compared to clinically used drugs.

*In vivo*, BALB/c mice were injected intraperitoneally with 250 tachyzoïtes of the 76K strain, an optimized dose of parasites to reach the chronic phase of infection, without lethality. Initially, we tested the dose of 2.5 mg/kg/day (50 μg per mouse) or 5 mg/kg/day (100 μg per mouse) of Imiquimod (65), every other day (Experimental scheme presented in Figure 1B). Transcriptional expression of SAG-1 was used as a surrogate to evaluate tachyzoïte dissemination to the spleens of mice. Consistent with the *in vitro* effect of Imiquimod on the tachyzoïte stages, we observed a significant decrease of SAG-1 transcripts in the spleen of treated mice (Figure 1B). Since 100 μg (5 mg/kg/day) resulted in similar outcome as 50 μg (2.5 mg/kg/day) (Figure 1B), the dose of 50 μg (2.5 mg/kg/day) was adopted throughout the study.

Host immune response to *Toxoplasma* infection is dependent on the parasite genotype. Intraperitoneal injection with tachyzoïtes of Type I strains leads to the recruitment of monocytes, macrophages and DCs to the peritoneum as early as day 2 p.i., [reviewed in Sher et al. (52), Ehmen and Luder (66)]. These cells produce IL-12, and present *T. gondii* antigens to prime T cells. T cell migration to the site of infection, peaks at day 6 p.i., leading to high systemic levels of IFN-γ required to control AT [reviewed in Sher et al. (52), Jordan et al. (67)]. Infections with type II strains lead to higher host cell migration rates and are more studied in the brains of infected mice (68). BALB/c mice were intraperitoneally injected with 1,000 tachyzoïtes of the 76K strain. Mice were treated with Imiquimod or sulfadiazine, on two consecutive days, and sacrificed on day 4 p.i. (Experimental scheme presented in Figure 1C). Immune cell subtypes were assessed by flow cytometry using CD11b which is expressed on the surface of monocytes, neutrophils, natural killer cells, granulocytes and macrophages, and CD3 marker which is expressed on T cells. The change in the number of CD11b^+^ cells in the peritoneum, was non-statistically significant upon treatment with Imiquimod or sulfadiazine. However, Imiquimod treatment selectively resulted in a significant increase of the CD3^+^ cell population (Figure 1C, Supplementary Figures 1A,B). In the spleen, Imiquimod and sulfadiazine significantly increased the number of CD11b^+^ cells, while the increase of CD3^+^ cells was specific to Imiquimod (Figure 1C). The transcriptional upregulation of IFN-γ, but not IL-12, at both sites (Figure 1D), supports a role for Imiquimod in a faster adaptive T immune response, potentially explaining the significant lower tachyzoïte burden observed during AT (Figure 1E).

### Treatment With Imiquimod During Acute Toxoplasmosis Reduces the Number of Bradyzoïte Cysts in the Brain and Impairs Their Infectivity

**Figure 2:**
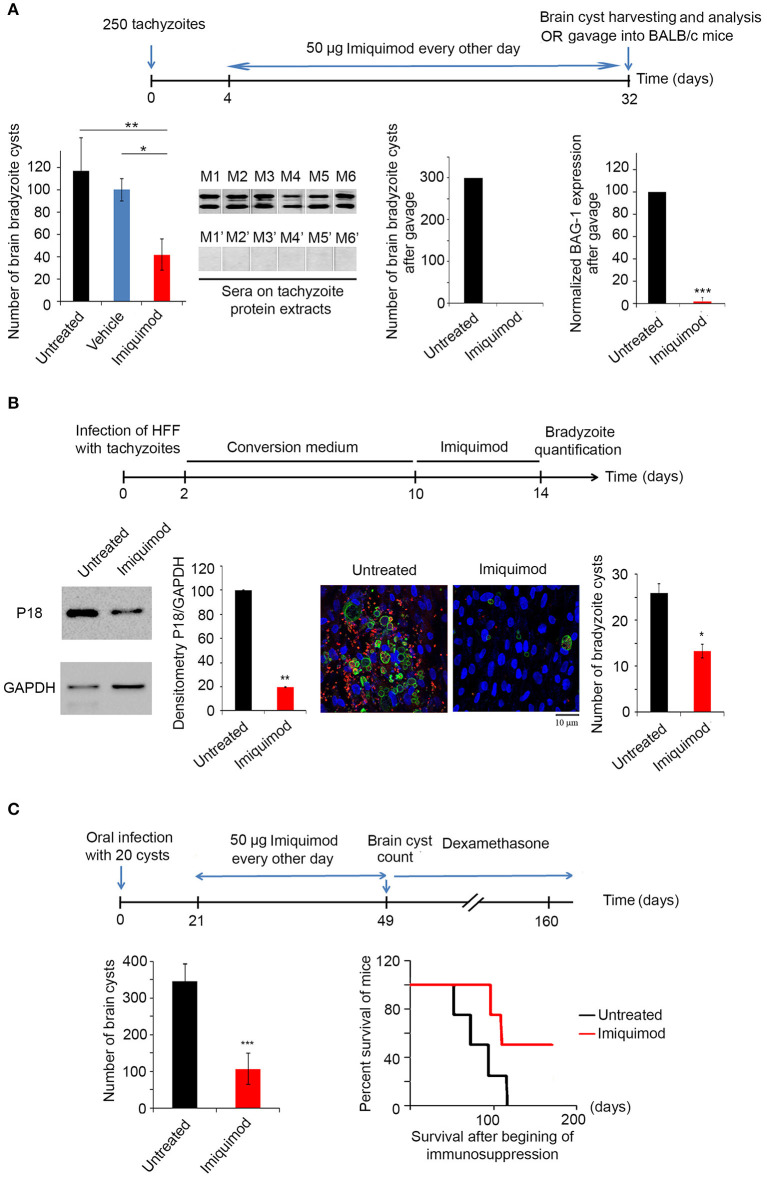
Imiquimod exhibits a potent effect on chronic toxoplasmosis. **(A)** Timeline schedule for assessment the effect of Imiquimod on the conversion from AT to CT in BALB/c mice. Briefly, on day 0, BALB/c mice were injected with 250 tachyzoïtes/mouse of 76K. On day 4, 50 μg/mouse of Imiquimod was administered every other day, during 4 weeks. At day 32, brains were harvested for cysts quantification or for gavage into new BALB/c mice. Cyst count was performed following percoll extraction (First panel, ten mice per condition). The Anova one way test was performed to validate significance. *, **, and *** indicate *p*-values ≤ 0.05; 0.01; and 0.001, respectively. *P*-values less than 0.05 were considered significant. Western blot using sera from mice orally infected with 20 cysts from untreated mice (M1 to M6), or from mice orally infected with 20 cysts from Imiquiod-treated mice (M1' to M6') against tachyzoïte protein extracts (Second panel). Cyst count for BALB/c orally infected with 20 cysts from brains of treated mice with Imiquimod (Third panel, ten mice per condition), and Quantitative Real-Time PCR for BAG-1 (Fourth panel, ten mice per condition) are shown as indicated. BAG-1 expression was normalized to GAPDH. The untreated group represent the average of different mice all considered as 100 percent **(B)** Timeline schedule for assessment of bradyzoïte cysts number *in vitro* following treatment with 1 μm of Imiquimod. Western Blot analysis for P18 expression and corresponding densitometry (left panel) following treatment of *in vitro* switch from tachyzoïtes to bradyzoïtes. The quantification of cysts normalized per cell number was confirmed by immunofluorescence assay (right panel), using a biotinylated lectin (green), with specific binding to a selectin on the cyst wall, the bradyzoïte marker P34 (red) and the Dapi staining for the nucleus of cells. Fifty cells were counted per field. The results depict one representative experiment among at least three independent ones. Number of cysts was determined in 50 independent fields per condition. **(C)** Timeline schedule for assessment of the effect of Imiquimod on a developed CT. Chronically infected mice were treated with Imiquimod starting day 21 until day 49. A group of mice was sacrificed for cyst quantification (ten mice per condition), while the survival of another group was assessed following immunosuppression with dexamethasone (eight mice per condition). On day 49, brains of BALB/c mice were harvested and cyst count was performed (left panel). The t-test was performed to validate significance. *, **, and *** indicate *p*-values ≤ 0.05; 0.01; and 0.001, respectively. *P*-values less than 0.05 were considered significant. Kaplan Meier (right panel) showing the survival of untreated (black line) and Imiquimod-treated (red line) BALB/c mice following dexamethasone immunosuppression (four mice per condition, one representative experiment of two independent ones).

We then tested the *in vivo* potency of Imiquimod on the establishment of CT, following treatment of mice during AT. BALB/c mice were intraperitoneally injected with 250 tachyzoïtes of the 76K strain. Imiquimod (50 μg) was administered every other day, until day 32 (Experimental scheme presented in Figure 2A). AT was verified seven days p.i. by immune reactivity of infected mice on tachyzoïte extracts (69). Thirty-two days p.i., bradyzoïte brain cysts were counted. Treatment with Imiquimod during AT, drastically reduced the number of brain cysts (120 to 30) (Figure 2A, first panel). The ability of the remaining cysts to induce a new infection was evaluated by orally infecting naïve mice with 20 cysts harvested from brains of either untreated or treated mice. Similarly, on day 7 post-oral infection, the acute phase was verified by immune reactivity of infected mice on tachyzoïte protein extracts (69). Reactivity of the serum of mice infected with cysts derived from brains of Imiquimod-treated mice was abolished (Figure 2A, second panel), suggesting an effect of Imiquimod on either the viability of cysts or the conversion of bradyzoïtes to tachyzoïtes to establish a successful infection. At day 32 post-oral infection, brains of infected mice with cysts derived from Imiquimod-treated animals were free of cysts (Figure 2A, third panel). This was further confirmed by the transcriptional expression of the bradyzoïte specific gene *BAG-1* (Figure 2A, fourth panel). These findings have far-reaching applications in blocking parasite transmission among hosts.

### Imiquimod Reduces the Number of Bradyzoïte Cysts and Abrogates Reactivation of Murine Chronic Toxoplasmosis

We then assessed the potency of Imiquimod on CT. *In vitro* interconversion from tachyzoïtes to bradyzoïtes was performed in HFF. On day 10, Imiquimod was added to cells infected with bradyzoïtes, at the dose of 1 μM and maintained for 4 days (Experimental scheme presented in Figure 2B). On day 14 p.i., treatment with Imiquimod significantly decreased protein levels of the abundantly expressed bradyzoïte protein P18 (58, 59), as compared to the untreated controls (Figure 2B). Similar results were obtained upon quantification of bradyzoïte cysts using a DBA specifically binding to a selectin on the cyst wall (57) (Figure 2B). These results demonstrate the potency of Imiquimod on the number of cysts *in vitro*.

CT is the most common form of toxoplasmosis (20) and correlates with several neuro-pathologies and cancers (11–13, 15, 16). Furthermore, CT reactivation may become life threatening in immunocompromised patients (18–20). In light of the lack of effective treatment options against CT, we assessed the effect of Imiquimod on chronically infected mice. BALB/c mice were infected with 250 tachyzoïtes of the 76K strain. The acute phase was verified 7 days p.i. by immune reactivity of infected mice on tachyzoïte protein extracts (69). Following establishment of CT, Imiquimod treatment was administered at the dose of 50 μg, every other day, from day 21 until day 49 p.i. (Experimental scheme presented in Figure 2C). Mice were monitored on a daily basis and exhibited normal mobility and ability to reach food and water, they did not lose weight and they had normal grooming behavior and normal hair coat, assuring that Imiquimod did not yield any adverse effects over the whole period of treatment. At day 49, brain cysts were harvested and counted (62, 63). The number of bradyzoïte cysts in Imiquimod-treated mice markedly decreased (Figure 2C, left panel). More importantly, the simulation of immunosuppression using dexamethasone drug resulted in a delayed reactivation in 50% of immunosuppressed mice while a total abrogation of reactivation was observed in the remaining 50% of mice (Figure 2C, right panel). These results present Imiquimod as a potent drug against CT and position it as a therapeutic candidate against reactivation of CT in immunosuppressed patients.

### Imiquimod Induces the Expression of Toll-Like Receptors-7, 11, and 12 in the Brains of Chronically Infected Mice

**Figure 3:**
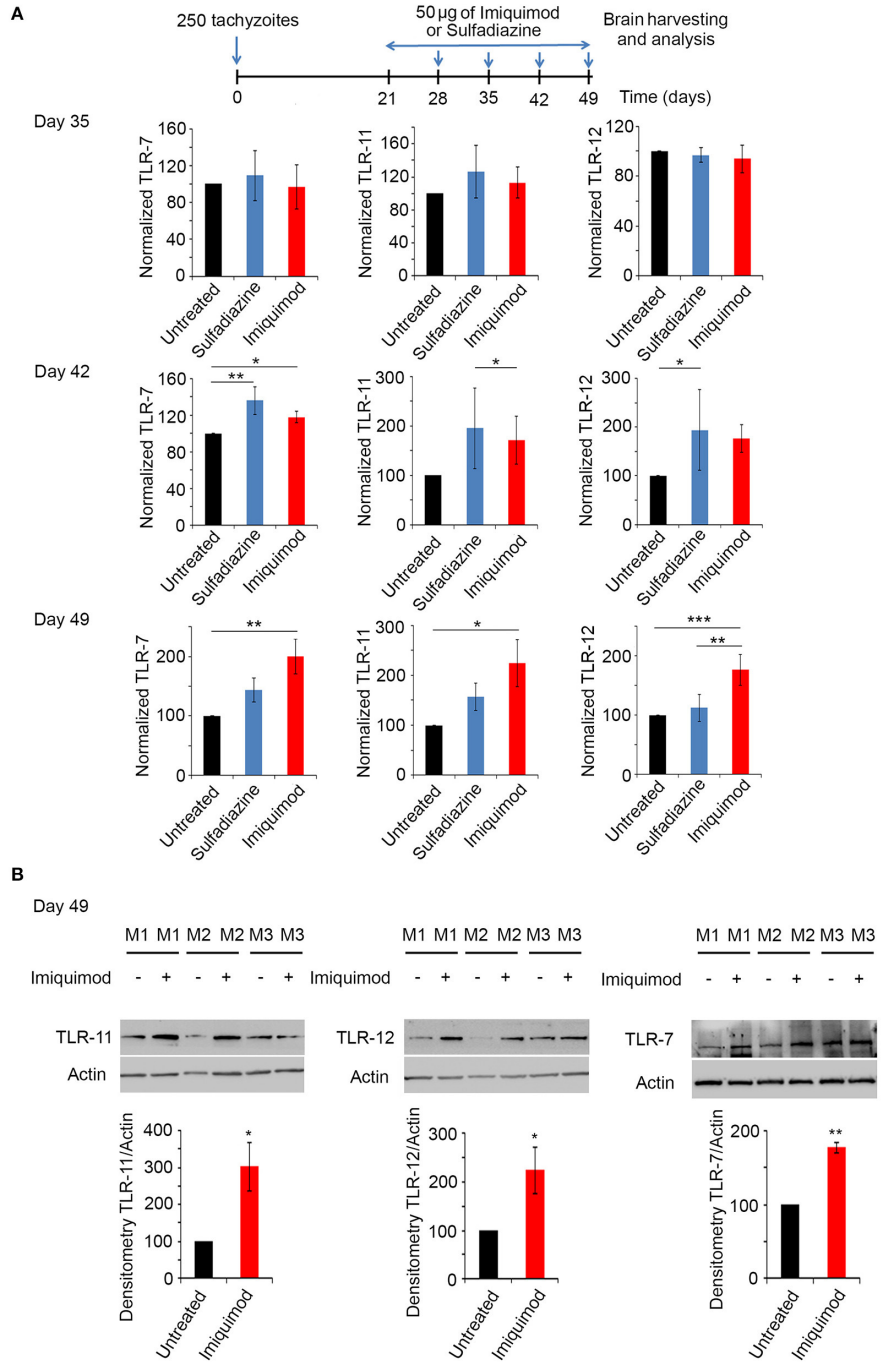
Imiquimod increases the expression levels of TLR-7,−11, and TLR-12. **(A)** Timeline schedule for assessment of TLR(s) profile in BALB/c mice. Briefly, BALB/c mice were injected with 250 tachyzoïtes/mouse of 76K. After treatment with 50 μg/mouse of Imiquimod or Sulfadiazine (200 mg/L in drinking water), mice were sacrificed at days 35, 42, 49, respectively. Quantitative Real-Time PCR for TLR-11, TLR-12, and TLR-7 (five mice per condition) from brains of these mice. TLRs expression was normalized to GAPDH. The results are expressed as percentage of untreated control (±) SD. The Anova one-way test was performed to validate significance. *, **, and *** indicate *p*-values ≤ 0.05; 0.01; and 0.001, respectively. *P*-values less than 0.05 were considered significant. **(B)** Western Blot analysis for TLR-11 (left panel), TLR-12 (middle panel) and TLR-7 (right panel) (three representatives out of five mice per condition) and their corresponding densitometry in the brains of BALB/c mice, following 4 weeks of treatment with Imiquimod. The untreated group represent the average of different mice all considered as 100 percent.

Toll-Like Receptor (TLR) signaling is one of the first defense systems against infections in mammalian-induced innate immunity. Imiquimod is a recognized agonist of TLR-7 (34, 37, 40). Profilin, a well-characterized *T. gondii* PAMP, is essential for the recognition of parasite antigens by TLR-11 and 12, expressed in murine DCs and monocytes during AT (45, 47, 55, 70, 71). Chronically infected BALB/c mice were treated with 50 μg of Imiquimod every other day, from day 21 until day 49 p.i. (Experimental scheme presented in Figure 3A). Mice were sacrificed at days 35, 42 and 49, and TLR-7, 11, and 12 transcript levels were analyzed by real time PCR in the harvested brains. The transcriptional levels of these TLRs increased starting day 42 and reached the highest level at day 49 p.i. (Figure 3A). Hence, we assessed protein levels of these TLRs at day 49. Predictably, protein levels of TLR-7, 11, and 12 were significantly increased upon treatment of chronically infected mice with Imiquimod (Figure 3B). These results reinforce a role for Imiquimod in signaling through these TLRs in chronic murine toxoplasmosis.

**Figure 4:**
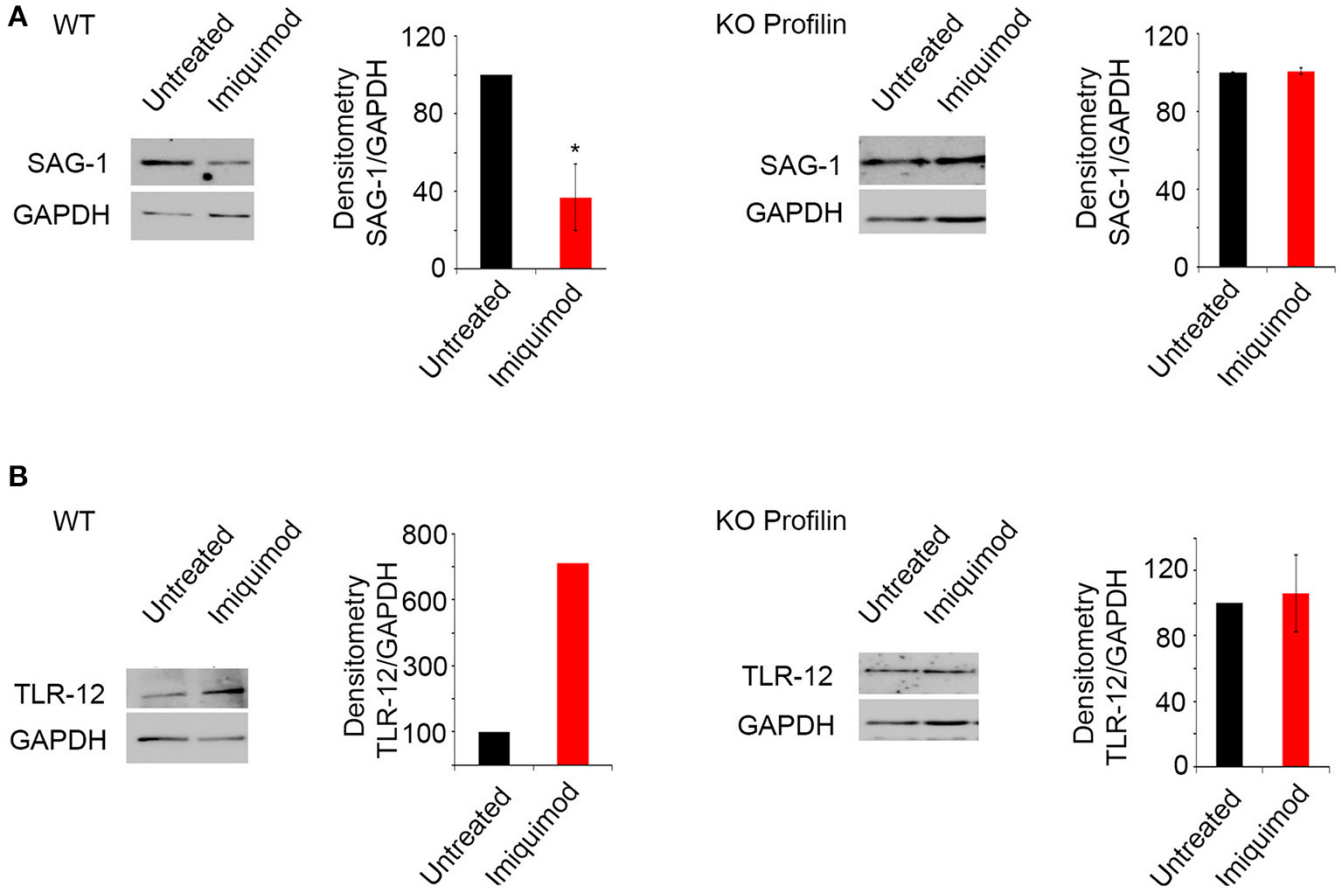
Imiquimod signals through Profilin/TLR-12 complex. Western Blot analysis and corresponding densitometry for SAG-1 **(A)**, TLR-12 **(B)** in the murine macrophages treated with 1 μm of Imiquimod following infection either with the wild type strain (left panel), or with the KO Profilin strain (right panel). The untreated group represent the average of different mice all considered as 100 percent. The results depict one representative experiment among at least three independent ones. *p-values ≤ 0.05, respectively. *P*-values less than 0.05 were considered significant.

Profilin is a parasitic PAMP, playing a major role in TLR binding and parasite invasion. It binds TLR-11 (47) and TLR-12 (46). Parasites lacking *Toxoplasma* Profilin, but expressing a *Plasmodium falciparum* profilin which restores the invasion and egress by the parasite, cannot activate TLR and are unable to induce TLR-11-dependent production of IL-12 both *in vitro* and *in vivo* (55). We evaluated the effect of Imiquimod on TRL11/12 signaling, in a *T. gondii* line depleted for *Toxoplasma* Profilin (ΔTg*PRFe*/Tg*PRFi*). We used murine macrophages which express TLR-11 and TLR-12 (42). Macrophages infected with ΔTg*PRFe*/Tg*PRFi* or RHTATi-1 at 1:3 parasite to cell ratio, were treated with 1 μM of Imiquimod for 24 h. Using SAG-1 protein as a marker for tachyzoïtes, Imiquimod had no effect on murine macrophages infected with ΔTg*PRFe*/Tg*PRFi* line (Figure 4A). Similarly, no effect was observed on TLR-12 protein expression in macrophages infected with the Knock-out profilin strain (Figure 4B). These results confirm the effect of Imiquimod through TLR11/12.

### Imiquimod Facilitates the Interconversion of Bradyzoïtes to Tachyzoïtes in the Brains of Chronically Infected Mice

**Figure 5:**
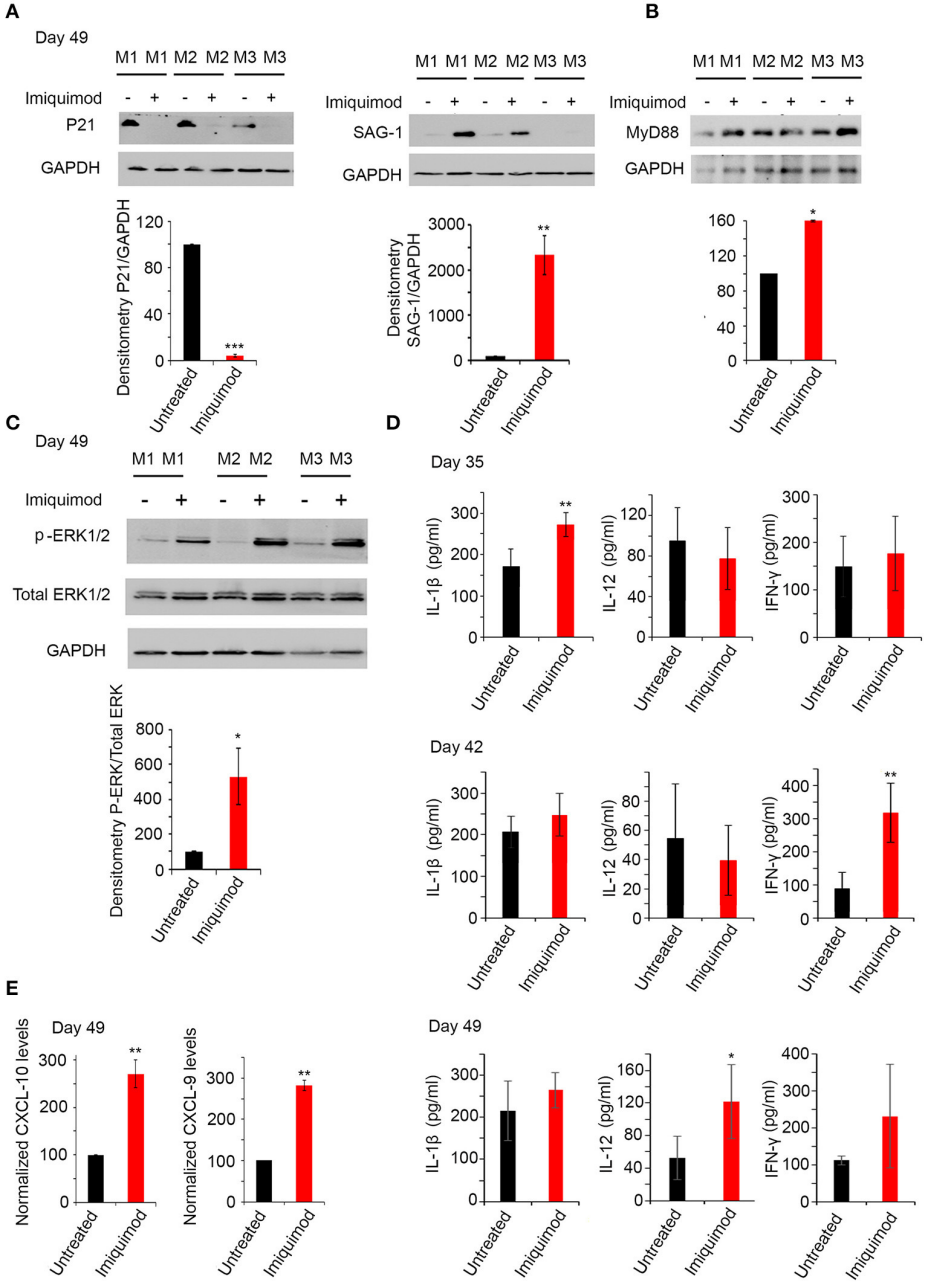
Imiquimod induces interconversion and activates the TLR-MyD88 signaling pathway. **(A)** Western Blot analysis for P21 (left panel), and SAG-1 (right panel) (three representatives out of five mice per condition) in total brain extracts of BALB/c mice sacrificed at day 49, following 4 weeks of treatment with Imiquimod, and their corresponding densitometry. **(B)** Western Blot analysis for MYD88 (three out of five representative mice per condition) in total brain extracts of BALB/c mice at day 49 following treatment with Imiquimod and their corresponding densitometry. The untreated group represent the average of different mice all considered as 100 percent. **(C)** Western Blot analysis for p-ERK1/2, and total ERK1/2 (three representatives out of five mice per condition) in total brain extracts of BALB/c mice at day 49, following 4 weeks of treatment with Imiquimod, and their corresponding densitometry. **(D)** ELISA showing the protein levels of IL-1β, IL-12, and IFN-γ, in the brains of chronically infected BALB/c mice with 76K and treated with 50 μg/mouse of Imiquimod for 2 weeks (upper panel), 3 weeks (middle panel), or 4 weeks (lower panel) (Five mice per condition, one representative experiment out of two). ELISA data were analyzed using Anova one-way test. *, **, and *** indicate *p*-values ≤ 0.05; 0.01; and 0.001, respectively. **(E)** Quantitative Real-Time PCR for CXCL-10 (left panel, five mice per condition) and CXCL-9 (right panel, five mice per condition), from total brain extracts of mice injected with 250 parasites of 76K, treated with Imiquimod for 4 weeks, and sacrificed on day 49. CXCL-9 and CXCL-10 expressions were normalized to GAPDH. The untreated group represent the average of different mice all considered as 100 percent. The results are expressed as percentage of untreated control (±) SD. The *t*-test was performed to validate significance. *, **, and *** indicate *p*-values ≤ 0.05; 0.01; and 0.001, respectively. *P*-values less than 0.05 were considered significant.

TLR-7, 11, and 12, known to be expressed in murine DCs, macrophages and monocytes are triggered by the tachyzoïte stage (51). We studied the potential effect of Imiquimod on interconversion from bradyzoïtes to tachyzoïtes. Remarkably, the protein levels of the late bradyzoïte specific marker P21 (58, 59), sharply decreased in brains of chronically infected mice, after 4 weeks of treatment with Imiquimod (Figure 5A, left panel). This decrease was concomitant with a significant upregulation in the protein levels of the tachyzoïte surface marker SAG-1 (58) (Figure 5A, right panel). These results implicate Imiquimod treatment in the interconversion from bradyzoïtes to tachyzoïtes in chronically infected mice.

### Imiquimod Signals Through TLR-MyD88 Pathway

MyD88 pathway is activated as a result of specific TLR signaling (49). Mice deficient in the adapter molecule MyD88 are acutely susceptible to toxoplasmosis (47). Consistent with TLR-7, 11 and 12 upregulation, MyD88 protein levels were upregulated following 4 weeks of treatment of chronically infected mice with Imiquimod (Figure 5B). A significant upregulation in p-ERK1/2, a hallmark of the activation of MyD88 pathway (72), was obtained in chronically infected mice at day 49 of treatment with Imiquimod (Figure 5C). Furthermore, Imiquimod treatment induced the production of IL-12, IL-1β, and IFN-γ proteins (Figure 5D), which are essential to mount a protective immune response against *T. gondii* infection (47, 51, 72). The temporal expression of these cytokines started with a significant increase of IL-1β at day 35, IFN-γ at day 42 and IL-12 at day 49. These results indicate that Imiquimod effect is presumably hastening the sequential recruitment of specific immune cells. This is supported by the higher transcript levels of Chemokine (C-X-C motif) ligand 9 (CXCL9) and Chemokine (C-X-C motif) ligand 10 (CXCL10) in brain homogenates of chronically-infected mice, upon treatment with Imiquimod (Figure 5E). These chemokines have a documented role in the recruitment of T cells to control *T. gondii* reactivated foci (73, 74).

## Discussion

Currently, a plethora of targeted therapies against toxoplasmosis [reviewed in Montazeri et al. (32)] are investigated. These are mostly effective against tachyzoïtes, and only few target bradyzoïtes (32). Ideally, an effective intervention must include, not only a treatment regimen, but also the ability to halt the dissemination of the parasite among hosts, and alleviate the burden of diseases associated with CT.

**Figure 6:**
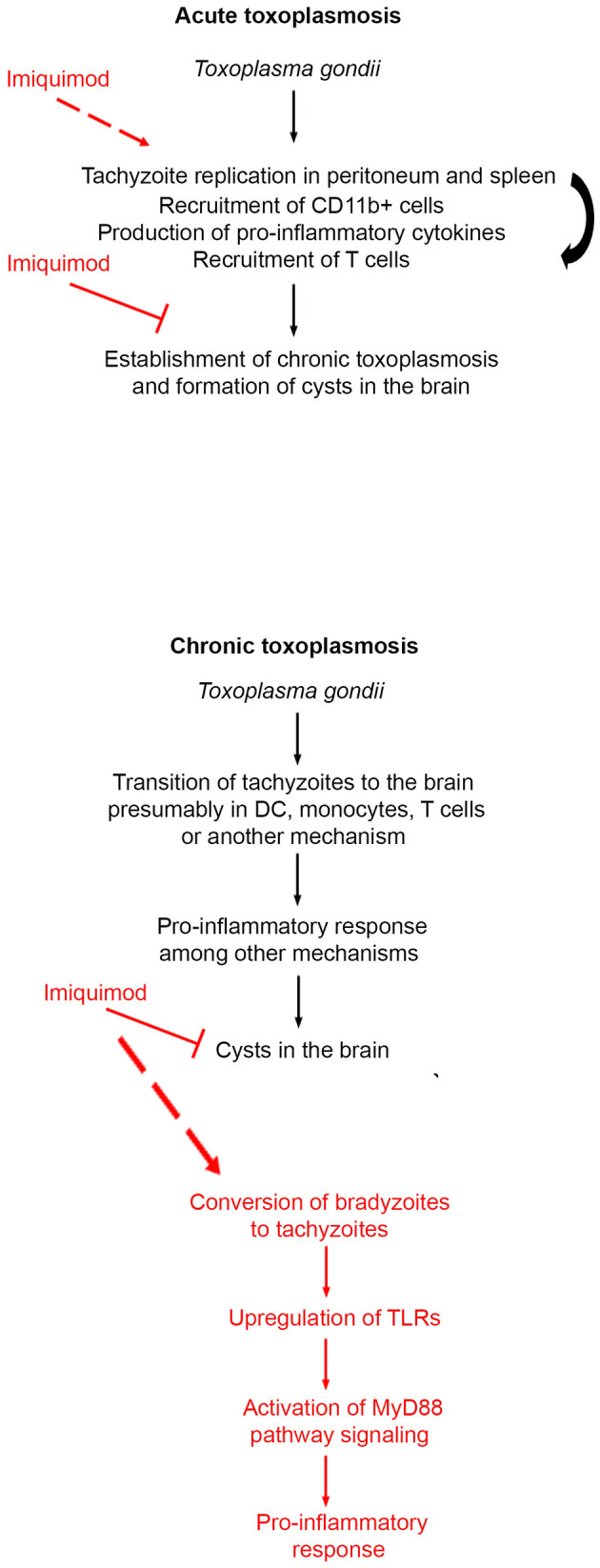
Schematic summary of the mechanism of action of Imiquimod on acute and chronic toxoplasmosis.

Imiquimod was identified as an effective treatment against some parasitic and viral infections (34–38). Furthermore, Imiquimod proved potent and safe when used as simple vaccine adjuvant in mice (75). In the context of toxoplasmosis, and using a type I virulent strain, Imiquimod showed promising prophylactic and therapeutic activities against acute toxoplasmosis in BALB/c mice (39). Yet, the virulent type I strains do not allow studying chronic toxoplasmosis, which the most common form of the disease in intermediate hosts, including humans. Imiquimod exerts its effect by modulating the host immune response through TLR signaling (40, 41). The reciprocal interplay between *T. gondii* and the host immune system determines the disease outcome and progression between AT and CT. In this study, we investigated the potency of the immune-modulatory drug Imiquimod, as a therapeutic modality, against acute and more importantly chronic toxoplasmosis. *In vitro*, Imiquimod led to a pronounced effect against tachyzoïtes in infected macrophages. This result is consistent with the recent work by (39). *In vivo*, Imiquimod reduced tachyzoïte burden in the spleen, likely as a consequence to the increase of CD11b positive innate immune cells. This treatment also resulted in an early recruitment of T cells, which are believed to control AT (68). Treatment with Imiquimod during AT, resulted in a drastic reduction in the number of brain cysts. Remarkably, a total abrogation of the capacity of the remaining cysts to reactivate was observed (Figure 6). The significance of our findings can positively impact domestic animal stock livelihood and veterinary medicine, where AT is responsible for an important economic loss (76).

CT is the most common form of toxoplasmosis (20). Even if CT was perceived as asymptomatic, its incidence correlates with several neuro-pathologies and cancers (11–13, 15, 16). We showed that treatment with Imiquimod, of an established CT, reduces the number of cysts in the brains, which have an important connotation in the context of diseases associated with CT. CT reactivation still poses a serious threat in immunocompromised patients (18–20). The fact that Imiquimod treatment increased the survival of immunosuppressed mice (Figure 6), supports a function for this drug in patients undergoing transplantation or any other immunosuppressive therapy.

Innate immunity represents the first line of defense against *T. gondii*. DCs and monocytes represent a major forefront manipulated by the parasite. This is in part due to the capacity of these cell types to mount a robust immune response and to the capacity of DCs to present antigens relevant to T cells recruitment and activity. As a consequence, the parasite-laden cells migrate to various organs, and reside in the brain as cysts (77). DCs recognize PAMPs via TLRs/MyD88 signaling (49). This signaling pathway is required for the immune protection against many infections, which in the absence of MyD88, are lethal (78). Two parasite PAMPs, Profilin and cyclophilin-18, play a role in TLR recognition. Profilin binds TLR-11 (47) and TLR-12 (46, 48), and enhances the production of IL-12 via MyD88-dependent pathway. TLR-11, in murine species, heterodimerizes with TLR-12, an important step for DC response and ultimately IL-12 production (46, 51). Our results demonstrated that Imiquimod increases the transcripts of TLR-11 and 12, most likely due to recruited DCs and monocytes. This in turn triggers MyD88-downstream signaling, the activation of MAPK, and the subsequent secretion of immune mediators including IL-1β, IL-12, and IFN-γ (Figure 6). IL-12 and IL-1β are pivotal in the recruitment of neutrophils and natural killer cells, which produce IFN-γ, whose levels are augmented by recruited T cells [reviewed in Yarovinsky (45), Sturge and Yarovinsky (79)]. In the brain, recruited *T. gondii*-infected DCs and monocytes participate, with astrocytes and microglia, to present antigens to activated CD4^+^ and CD8^+^ (80, 81) to control the infection (68). Imiquimod treatment increased the expression of CXCL9, which is associated with the recruitment of T cells into brain tachyzoïte reactivated foci (73). This supports the recruitment and activation of T cells, hence controlling the Imiquimod-induced conversion to tachyzoïtes (Figure 6). Since Imiquimod induces interconversion between brain stages, it is likely contraindicated in immunocompromised patients, despite the total abrogation of reactivation in 50% of treated mice. However, these results create an important application in solid organ transplantation where pre-treatment of transplanted organs will reduce the chances of infection, especially that the risk of infection with *T. gondii*, in heart, liver, kidney, and bone marrow transplantation is still extremely high (82–84).

A strain depleted from Profilin is refractory to treatment with Imiquimod (Figure 4A), and TLR-12 expression levels in cells infected with this parasite line remained unchanged (Figure 4B). These results suggest that Imiquimod may enhance the binding of this parasite PAMP, to induce TLR-mediated MyD88 signaling. Although humans do not express TLR-11 or TLR-12, human monocytes produce pro-inflammatory cytokines in response to *T. gondii* infection, suggesting that different TLRs are involved in humans, which produce IL-12 in antigen-presenting cells (51). It has been described that parasite recognition by human intracellular TLRs (TLR3, 7 and 9) facilitates resistance to toxoplasmic infection and activation of monocytes and DCs (51, 52). A study showed that the human TLR5 may have a similar role to the mouse TLR-11, in activating cytokine production (85). Our results showed that Imiquimod treatment, not surprisingly, leads to the upregulation of TLR-7 known to recognize ribonucleic acids (86), and to induce the production of inflammatory cytokines through MyD88 signaling (87, 88). The Imiquimod induced TLR-7 upregulation, in conjunction with TLR11 and 12, results in MyD88 signaling and cytokine production. This plausible mechanism can be exploited to treat CT and lessen the burden of associated diseases in humans.

Cyclophilin-18 is another parasitic PAMP, recognized by both mouse and human C-C chemokine receptor type 5 (CCR5) (89) and enhances the proliferation and migration of macrophages and spleen cells (mainly T lymphocytes), to the site of infection (90). The efficient recruitment of T cells to both peritoneum and spleen during AT, may implicate cyclophilin-18. It would be interesting to investigate the role of Cyclophilin-18 in this process.

Treatment with Imiquimod during AT, reduced the number of cysts upon establishment of CT, and that the remaining bradyzoïtes failed to convert to tachyzoïtes following oral infection. This result may serve as basis to treat AT infection in both humans and animals. Imiquimod treatment has positive implications in health outcomes of immunocompetent patients, where CT correlates with several neuro-pathologies and cancers. Furthermore, targeting bradyzoïte cysts, in brains and skeletal muscles, interferes with parasite survival, and persistence in intermediate hosts, as well as with the transmission between intermediate hosts and/or definitive hosts. Imiquimod, as supported by our data, stands to become novel therapy in *T. gondii* related diseases, since its performance exceeded that obtained from current gold standard treatment.

## Data Availability Statement

The original contributions presented in the study are included in the article/Supplementary Material, further inquiries can be directed to the corresponding author/s.

## Ethics Statement

The animal study was reviewed and approved by Institutional Animal Care and Utilization Committee (IACUC) of the American University of Beirut (AUB) (Permit Number: #18-02-461).

## Author Contributions

MH and RN performed experiments. CD-M, PB, J-FD, ME, and HE analyzed results. MH, ME, and HE made the Figures. HE designed the research. HE and ME wrote the paper. All authors contributed to the article and approved the submitted version.

## Conflict of Interest

The authors declare that the research was conducted in the absence of any commercial or financial relationships that could be construed as a potential conflict of interest.
