# Supplementary material for: Imiquimod Targets Toxoplasmosis Through Modulating Host Toll-Like Receptor-MyD88 Signaling
Source: Front Immunol. 2021 Mar 9;12:629917. doi: 10.3389/fimmu.2021.629917 (PMC7986122; doi:10.3389/fimmu.2021.629917)
Supplement: Supplementary file 1 [file Table_1.DOCX]

**
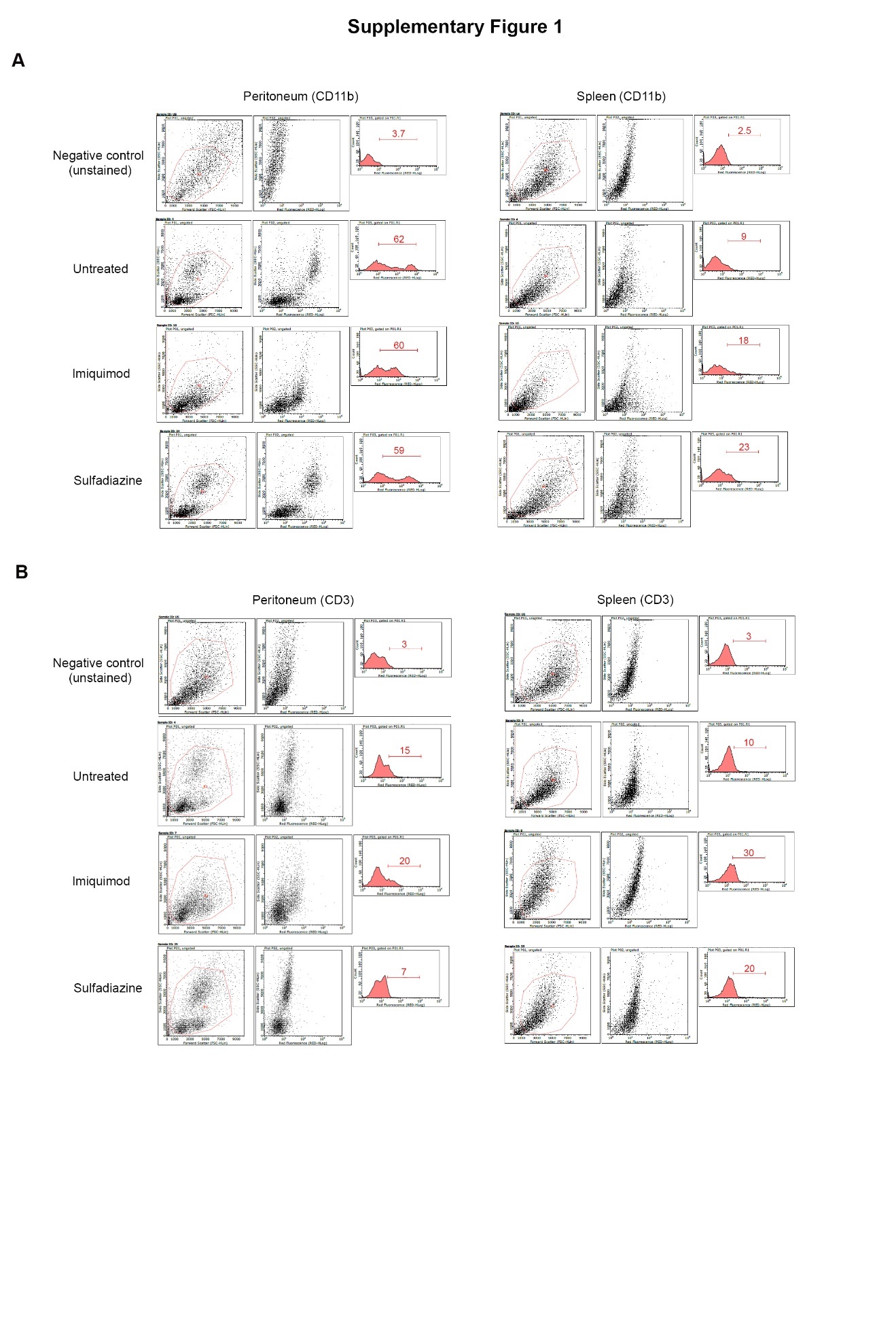
**

**Supplementary Figure 1.** On day 0, BALB/c mice were injected with 1000 tachyzoïtes/mouse of 76K (five mice per condition). On days 2 and 3 mice were treated either with Imiquimod (2.5mg/kg/day) or with sulfadiazine (200mg/L in drinking water). At day 4, spleens and peritoneal lavage were harvested and stained with anti-CD11b or anti-CD3, directly conjugated to Phycoerythrin (PE). Labelled samples were washed twice and 5000 events were acquired using a Guava flow cytometer easy Cyte ^TM^ 8 (Millipore). **(A)** Representative flow cytometry scatter dot plots of CD11b-PE expression in cells derived from the peritoneum (left Panel) or from spleen (right panel) of mice untreated or treated with imiquimod and sulfadiazine. Debris and dead cells were gated out by FSC versus SSC gating (P1). The percentage of CD11b+ population was determined based on the autofluorescence of unstained cells on the red channel. **(B)** Representative flow cytometry scatter dot plots of CD3-PE expression in cells derived from the peritoneum (left Panel) or from spleen (right panel) of mice untreated or treated with imiquimod and sulfadiazine. Debris and dead cells were gated out by FSC versus SSC gating (P1). The percentage of CD3^+^ population was determined based on the autofluorescence of unstained cells on the red channel.
